# Growth-promoting function of the cGAS-STING pathway in triple-negative breast cancer cells

**DOI:** 10.3389/fonc.2022.851795

**Published:** 2022-08-03

**Authors:** Liang-Chih Liu, Yi-Chun Shen, Yuan-Liang Wang, Wan-Rong Wu, Ling-Chu Chang, Ya-Huey Chen, Chuan-Chun Lee, Shao-Chun Wang

**Affiliations:** ^1^ Department of Medicine, College of Medicine, China Medical University, Taichung, Taiwan; ^2^ Department of Surgery, China Medical University Hospital, Taichung, Taiwan; ^3^ Graduate Institute of Biomedical Sciences, College of Medicine, China Medical University, Taichung, Taiwan; ^4^ Research Center for Cancer Biology, China Medical University, Taichung, Taiwan; ^5^ Center for Molecular Medicine, China Medical University Hospital, Taichung, Taiwan; ^6^ Cancer Biology and Drug Discovery Ph.D. Program, China Medical University, Taichung, Taiwan; ^7^ Department of Cancer Biology, University of Cincinnati, Cincinnati, OH, United States; ^8^ Department of Biotechnology, Asia University, Taichung, Taiwan

**Keywords:** triple-negative breast cancer, EGFR, cGAS, STING, ssDNA, cGAMP, autophagy, patient-derived tumor organoid

## Abstract

The cGAS-STING axis is one of the key mechanisms guarding cells from pathogen invasion in the cytoplasmic compartment. Sensing of foreign DNA in the cytosol by the cGAS-STING axis triggers a stress cascade, culminating at stimulation of the protein kinase TBK1 and subsequently activation of inflammatory response. In cancer cells, aberrant metabolism of the genomic DNA induced by the hostile milieu of tumor microenvironment or stresses brought about by cancer therapeutics are the major causes of the presence of nuclear DNA in the cytosol, which subsequently triggers a stress response. However, how the advanced tumors perceive and tolerate the potentially detrimental effects of cytosolic DNA remains unclear. Here we show that growth limitation by serum starvation activated the cGAS-STING pathway in breast cancer cells, and inhibition of cGAS-STING resulted in cell death through an autophagy-dependent mechanism. These results suggest that, instead of being subject to growth inhibition, tumors exploit the cGAS-STING axis and turn it to a survival advantage in the stressful microenvironment, providing a new therapeutic opportunity against advanced cancer. Concomitant inhibition of the cGAS-STING axis and growth factor signaling mediated by the epidermal growth factor receptor (EGFR) synergistically suppressed the development of tumor organoids derived from primary tumor tissues of triple-negative breast cancer (TNBC). The current study unveils an unexpected function of the cGAS-STING axis in promoting cancer cell survival and the potential of developing the stress-responding pathway as a therapeutic target, meanwhile highlights the substantial concerns of enhancing the pathway’s activity as a means of anti-cancer treatment.

## Introduction

Dysregulated replication and damage repair of genomic DNA frequently occurs in proliferating cancer cells, leading to generation of aberrant DNA species such as single-stranded DNA (ssDNA) in the cytosol, which in turn activates the cyclic guanosine monophosphate-adenosine monophosphate synthase (cGAS) to produce the secondary messenger cytosolic 2’,3’-cyclic guanylate-adenylyl monophosphate (cGAMP) (1–6). Binding of cGAMP to STING (stimulator of interferon genes) facilitates recruitment of the adaptor protein to the endoplasmic reticulum and activation of the serine/threonine protein kinase TANK-binding kinase 1 (TBK1). In consequence, the interferon regulatory factors (IRF3/7) are activated through phosphorylation by TBK1 which promotes their nuclear entry for transcriptional activation of the inflammatory type I interferons and subsequent anti-tumor immunity (3). Thus, the cGAS-STING-TBK1 DNA sensing cascade is traditionally considered as a measure of tumor suppression.

Tumor development usually takes place in hostile poorly vascularized environments, with limited supply of nutrients and oxygen, compounded by immune surveillance as well as the bombardments of chemotherapies and targeted therapies. The stressed milieu fosters deregulated proliferation and genomic instability in cancer cells, in which the aberrant genomic DNA metabolism causes the presence of spontaneous intermediate nucleic acids derived from the nuclear DNA in the cytosol of the stressed cancer cells (7). It is traditionally conceived that under this condition the constant activation of cGAS by the cytosolic DNA should suppress tumor development through the induction of tumor-opposing inflammatory response. However, in reality spontaneous production of type I interferons is rare in human cancer (8), and loss-of-function mutations in the cGAS and STING genes are also scarce in human cancers (9). These observations would speculate that the cGAS-STING axis may somehow contribute to tumor progression while going along with the chronical stresses.

In the current study it is demonstrated that serum starvation or inhibition of the growth signaling of the receptor tyrosine kinase (RTK) epidermal growth factor receptor (EGFR) induces cytosolic ssDNA and activation of the cGAS-STING-TBK1 pathway which conveys a survival advantage in the cancer cells. Pharmacological inhibition or depletion of cGAS by RNA interference resulted in growth suppression. Evidence is provided to support that autophagy, but not other forms of cell death, is the main mechanism leading to suppression of cancer cells. The growth suppression can be recapitulated in tumor organoids derived from the primary tumors of breast cancer patients.

## Materials and methods

### Reagents and cell lines

MDA-MB-231 cells were maintained in the media of DMEM/F12 with 10% FBS, and SUM159 cells were cultured in DMEM/F12 with 10% FBS containing hydrocoretisone (1 μg/ml). The following antibodies were purchased: cGAS (Arigo Biolaboratories); AMPKα, pT172-AMPKα, and pS172-TBK1 (Cell Signaling Technology); TBK1 (Novus Biologicals); β-actin (Santa Cruz Biotechnology), mouse and rabbit secondary antibody-conjugated horseradish peroxidase (Jackson ImmunoResearch); and secondary antibodies for immunofluorescence staining (Invitrogen). RU.521 was purchased from *In vivo*Gen; propidium Iodide, 2’,3’-cGAMP, pan caspase inhibitor (Z-VAD-FMK) and autophagey inhibitor (3-MA and chloroquine) were purchased from Sigma Aldrich; inhibitors of necrosis and ferroptosis (necrostatin-1 and ferrostatin-1) were purchased from Cayman Chemicals. The shRNA clones of ATG5 (TRCN0000330394), cGAS (TRCN0000149984), and STING (TRCN0000134594) were obtained from the RNA Technology Platform and Gene Manipulation Core of the Academia Sinica in Taiwan.

### Immunofluorescence staining

MDA-MB-231 and SUM159 cells were seeded on glass coverslips for 20 hr, and then treated with either the control vehicle or RU.521 in the presence and absence of serum for 24 hr. Cells were washed with 1 × PBS, and fixed by 4% paraformaldehyde at room temperature for 20 min before being permeabilized by 0.01% Triton X-100 at room temperature for 10 min. Goat serum (5%) in 1 × PBS was applied to the slides to blocked non-specific site at room temperature for 1 hr. The blocked cells were then stained with primary antibodies against pS172-TBK1 or TBK1 for 16 hr at 4°C, and incubated with the secondary antibodies conjugated with fluorescence for 1hr at room temperature. The flourescent images were captured by fluorescence microscopy at a magnification of 630×. The fluorescence intensity (IF) was quantitied by ImageJ and normalized by the number of cells. For each staining experiment, three independent fields were examined and quantitated. The statistical significance of the results was determined by Student’s t test.

### Detection of cell survival by crystal violet staining

MDA-MB-231 and SUM159 cells were seeded in 96-well tissue plate at a cell density of 3x10^3^ cells for 20 hr, and then treated either with vehicle (DMSO) or different concentrations of RU.521 under serum-replete or serum-depleted condition for 48 hr. The treated cells were washed with 1x PBS, and fixed with 4% paraformaldehyde for 10 min at room temperature. Cells were then stained by 100 μl crystal violet (5 mg/ml in 2% ethanol) at room temperature for 10 min. The stained plate was washed clean, and air-dried at room temperature. The crystal violet dye was solubilized by adding 200 μl 1% SDS and incubation for 30 min at room temperature. The amount of the dye was measured by absorbance at 590 nm.

### Lentiviral shRNA amplification and transduction

The lentiviral clones of cGAS and STING shRNA were purchased from the RNA Technology Platform and Gene Manipulation Core of the Academia Sinica in Taiwan. The shRNA lentiviral vector was transfected to HEK293FT cells with the lentiviral packaging plasmids (VSV-G and Δ8.91) using lipofectamine 2000 (Thermo Fisher). The virus-containing media were collected at the time points of 48 and 72 hrs, and cell debris was removed by centrifugation at 800g for 10 min. The cleared media was aliqouted and stored at -80°C. MDA-MB-231 and SUM159 cells were plated in 24-well tissue plates at a low density (3x10^4^ cells) and incubated for 20 hr. Cells were infected with 500 ul of the virus-containing medium in fresh growth medium in the presence of polybrene (8 μg/ml) for 24 hr. After puromycin selection for 3 day, the infected cells were amplified, and the knockdown efficiency was determined by Western blotting using specific antibodies (10).

### Detection of cell cycle by flow cytometry

Detached cells were washed by ice-cold 1× PBS and fixed in 70% ethanol at -20°C for 16 hr. The fixed cells were spun down at 1400 rpm for 5 min and washed by ice-cold 1× PBS twice. Washed cells were ressuspended in PI buffer (20 μg/ml propidium iodide, 0.1% Triton X-100, 10 μg/ml RNase) and stained for 30 min at room temperature. Propidium iodide was then washed by centrifugation and cells were resuspended in ice-cold 1× PBS. Cell cycle distribution was then analyzied by BD FACSVerse™ flow cytometer (BD Biosciences).

### Detection of cell apoptosis by 7-AAD/Annexin V staining and flow cytometry

MDA-MB-231 and SUM159 cells were plated in 6-well tissue plates (1x10^5^ cells) for 20 hr, and then cultured in serum-replete and serum-depleted condition for 48 hr. The detached cells were washed by ice-cold 1x PBS and stained with 7-AAD (7-aminoactinomycin D) (Thermo Fisher) and the anti-Annexin V antibody (BioLegend) for 30 min at room temperature. The apoptotic cell population was analyzed by FACSVerse flow cytometer (BD Biosciences).

### Western blotting analysis

Cells were harvested in RIPA buffer (150 mM NaCl, 1% NP40, 0.5% sodium deoxycholate (DOC), 0.1% SDS, 50 mM Tris pH 7.5, 10 μg/ml Aprotinin, 5 mM PMSF, 25 mM NaF, 2 mM Na_3_VO_4_) and sonicated for 10 min at 4°C. Protein concentrations were measured by a protein assay kit (Bio-Rad). Cell lysate were separated through SDS-PAGE gels and transferred to PVDF membranes (Immobilon). The membranes were washed by TBST (0.05% Tween 20, 1.37 M NaCl, 27 mM KCl, 190 mM Tris-base, pH 7.4) and blocked by 5% nonfat milk for 1 hr at room temperature before incubating with primary antibodies for 16 hr at 4°C, followed by incubating with secondary antibodies conjugated with HRP for 1 hr at room temperature. The singals were visualized by using a chemoluminescience kit (Advansta) and captured by the ChemiDoc™ Touch imaging system (Bio-Rad).

### Detection of cellular ssDNA by OliGreen

Cells planted in 24-well tissue plates (3×10^4^ cells/well) for 16 hr were stained with OliGeen (Invitrogen) and Hoechst (Invitrogen) for 30 min at 37°C. The fluorescence images were captured by fluorescence microscopy at a magnification of 400×.

### Combination index (CI) assessment of RU.521 and afatinib

The drug combination index of RU.521 and aftatinib was calculated by the formula CI=(D)_1_/(Dx)_1_+(D)_2_/(Dx)_2_, where CI < 1, CI = 1, CI > 1 indicates synergistic, additive, and antagonistic, respectively (11).

### Patient-derived tumor organoids

Breast tumor tissue specimens were obtained with approval of the Institutional Review Board (IRB) (CMUH105-REC1-064(CR-5)). All human subject studies follow the declaration of Helsinki principles. Organoid #1 and #2 were derived from the tumros of two breast cancer patients, 109 (ER^+^, PgR^-^, Her2 equivocal) and 099 (ER^-^, PgR^-^, Her2^-^, referred to as a TNBC tumor, respectively. Briefly, fresh breast cancer tissues were minced into pieces of 1-3 mm^3^. The minced tissues were washed by the AdDF+++ medium (advanced DMEM/F12 medium containing Glutamax x1, HEPES 10 mM, and 1% antibiotics), and the tissue pellets were digested by collagenase (1-2 mg/ml; Sigma, C9407) while incubating at 37°C for 1 hr with gental shaking. The suspension were collected and strained through a filter with a pore size of 100 μm in diameter. The retained tissue blockes were collected by washing with 5 ml of AdDF+++ medium containing 2% FBS and centrifuged at 400 g. The tissue pellet was wahsed again using the same buffer, resuspended, and embeded in 40 ul of reduced growth factor cold Cultrex BME type 2 (Trevigen, 3533-010-02), each in a well of a 24-well plate and allowed to solidify at 37°C for 20 mins. The culture was then incubated in 400 μl of BC organoid medium in a humidified incubator at 37°C with 5% CO_2_ (12).

### Statistical analysis

All experiments were performed at least three times and data were presented as mean ± standard deviation (SD). Statistical significance analysis was performed by Student’s t-test.

## Results

### Serum starvation induces cytosolic ssDNA

**Figure 1:**
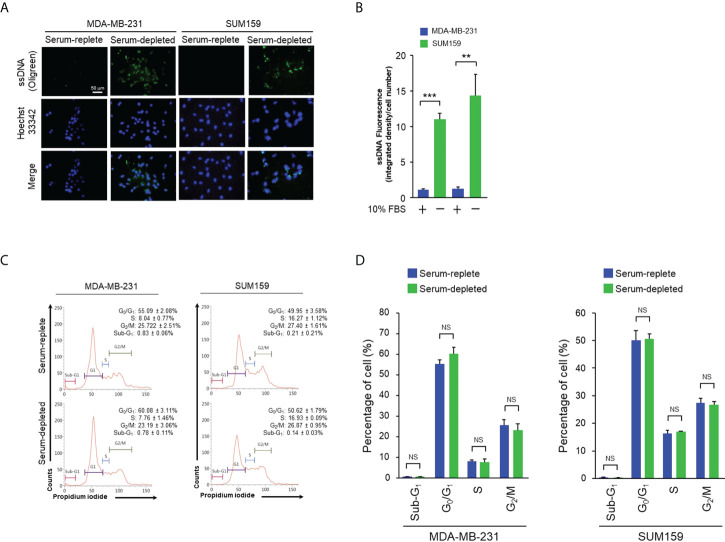
Serum depletion induced cytosolic ssDNA accumulation in TNBC cells. **(A)**, MDA-MB-231 and SUM159 cells were cultured in normal or serum-depleted media for 24 hr. The production of ssDNA expression was detected by OliGreen staining. The nuclei were counterstained with Hoechst 33342. Fluorescent images were captured at 400x magnification. Bar, 50 μm. **(B)**, The images in **(A)** were quantitated and plotted. The data of three independent results are shown as means ± SD (standard deviation) and the statistical significance was calculated by Student’s t-test. **, p < 0.01; ***, p < 0.001. C, The indicated cells were cultured as described in **(A)** for 24 hr, fixed, and stained with propidium iodide. Cell cycle profiles were analyzed by flow cytometry. The proportions of cells in sub-G_1_, G_0_/G_1_, S and G_2_/M were indicated with corresponding standard deviation. **(D)**, The results of three independent biological repeats of cell cycle profiling as described in **(C)** were plotted with means ± SD. Statistical significance was calculated by Student’s test. NS, non-significant with the p values greater than 0.05.

**Figure 2:**
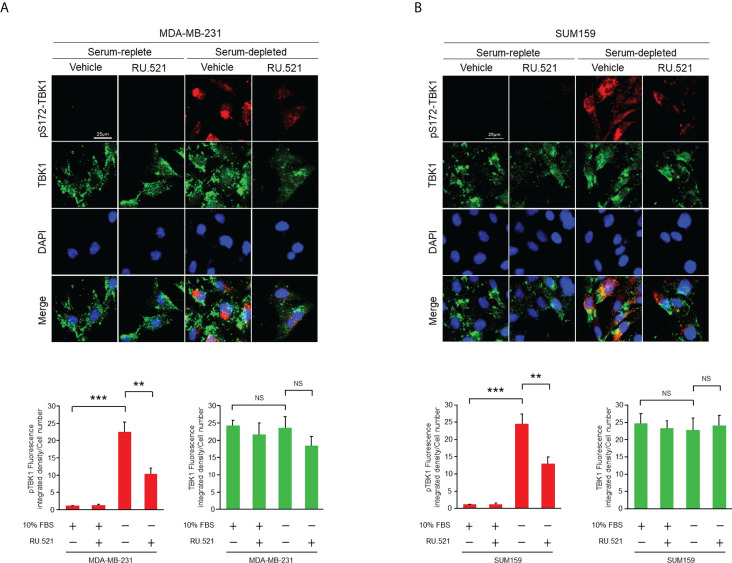
The cGAS-STING pathway of TNBC cells was activated by serum depletion which was suppressed by treatment with RU.521. MDA-MB-231 cells **(A)** and SUM159 cells **(B)** were grown in normal or serum-depleted media in the presence or absence of RU.521 (10 μM) for 24 hr. Total TBK1 and pSer172 TBK1 were detected by immunofluorescence staining with the corresponding antibodies. Fluorescent images were captured at 630x magnification. The nuclei were counterstained by DAPI. Bar, 25 μm. The images were quantitated and plotted with three independent results are shown as means ± SD, and the statistical significance was calculated by Student’s t-test. **, p < 0.01, ***, p < 0.001. NS, non-significant with the p values greater than 0.05.

Prominent accumulation of ssDNA was observed in the cytosol of MDA-MB-231 and SUM159, both are human triple-negative breast cancer (TNBC) cell lines, cultured in serum-free media for 24 hr by staining with the ssDNA-binding fluorescent dye OliGreen (**Figures 1A, B**). Under this growth condition, no significant cell death or aberrant cell cycle progression was detected as shown by flow cytometry analyses (**Figures 1C, D**), indicating that the spontaneous cytosolic ssDNA is not simply the consequence of growth impairment. To test whether the ssDNA is associated with enhanced cGAS-STING activity, MDA-MB-231 and SUM159 cells were cultured in normal or serum-free media in the presence and absence of the pharmacological compound RU.521, a potent investigational inhibitor of cGAS to be used for treating inflammatory symptoms of autoimmune diseases (13, 14). Western blotting analysis confirmed similar expression levels of the cGAS protein in both serum-replete and -stripped media in the presence and absence of RU.521. Similarly, the expression of STING was not affected by RU.521 in normal serum (**Supplementary Figure 1**). However, STING expression was downregulated by the treatment of RU.521 in serum-depleted media. Furthermore, expression of total and the activated TBK1, indicated by its phosphorylation at Ser172, were assessed by immunofluorescence. The results showed that TBK1 was activated in serum-free media, which can be inhibited by treatment with RU.521 (**Figures 2A, B**). This was confirmed by Western blotting analysis showing that serum depletion induced the activation of TBK1 (pS172-TBK1) and IRF3 (pS396-IRF3), as well as the production of the downstream type I interferons IFNα and IFNβ; conversely, treatment with RU.521 cancelled the activation of the cascade (**Supplementary Figure 2**).

### Inhibition of the cGAS-STING axis suppresses cell viability in serum-depleted media

**Figure 3:**
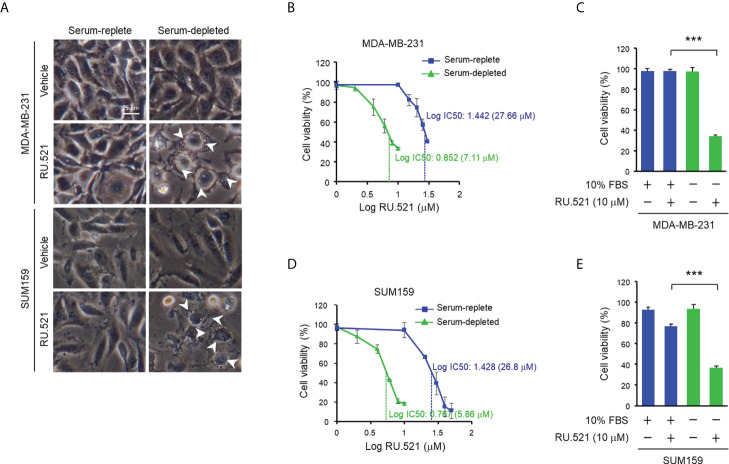
Treatment with RU.521 abrogated viability of TNBC cells cultured in serum-depleted media. **(A)**, MDA-MB-231 and SUM159 cells were cultured in normal growth media containing 10% FBS or serum-depleted media for 48 hr. Bright field images were taken at 400x magnification. White arrowheads indicate the cytoplasmic vacuoles. Bar, 25 μm. **(B–E)** MDA-MB-231 **(B, C)** and SUM159 **(D, E)** cells cultured in normal growth media containing 10% FBS (blue) or serum-depleted media (green) treated with different doses **(B, D)** and at the dose of 10 μM **(C, E)** of RU.521 for 48 hr. Cell viability was determined by crystal violet staining and detected by absorbance at 595 nm. The results of three independent experiments are shown as means ± SD. Statistical significance was calculated by Student’s t-test. ***, p < 0.001. The half maximal inhibitory concentration (IC_50_) of RU.521 for each cell line is indicated.

Given the induction of the ssDNA-cGAS-STING pathway in cancer cells cultured in serum-depleted media, it was speculated that the prolonged activation of the cGAS-STING axis in response to the stress of nutrient deprivation may have an impact on cell viability. To test this hypothesis, MDA-MB-231 and SUM159 cells were cultured in media containing normal (10%) or low (1%) amount of serum, or without serum (serum-depleted) for 48 hr. Consistent to the observation made after incubation of 24 hr (**Figure 1**), both cell lines showed prominent accumulation of ssDNA in the cytosol under this condition (**Supplementary Figures 3A, B** and **Supplementary Figures 4A, B**). On the other hand, staining for the double-stranded DNA (dsDNA) with the dye PicoGreen showed no difference in either normal or serum-stripped culture in the two cell lines (**Supplementary Figures 3C, D**). As expected, cell cycle analyses by flow cytometry after BrdU incorporation and 7-AAD staining showed increased G_1_ phase, and reduced S as well as G_2_/M phases in serum-depleted media (**Supplementary Figures 3E, F**). Less than 10% of cells were in the sub-G_1_ phase, suggesting a low level of cell death and that most cells remained alive. This was further confirmed by staining with 7-AAD and anti-annexin V in which less than 10% of apoptotic cells were detected in both serum-replete and serum-starved conditions (**Supplementary Figures 3G, H**). Consistently, the morphology of the MDA-MB-231 and SUM159 cells cultured in serum-depleted media maintained intact (**Figure 3A**). To test the role of the cGAS-STING axis, cells were treated with RU.521. The treatment induced accumulation of cytoplasmic vacuoles, a prominent feature consistent with the occurrence of autophagy (**Figure 3A**) (15). Reduction of viable cells by RU.521 was confirmed with the decrease of crystal violet staining after the treatment (**Figures 3B–E**). The half maximal growth inhibition concentration (IC_50_) of RU.521 to MDA-MB-231 was 27.7 μM in serum-replete media, which dropped to 7.1 μM in serum-depleted media (**Figures 3B, C**). Similarly, the IC_50_ of RU.521 to SUM159 cells was 26.8 μM in serum-replete media and 5.9 μM in serum-depleted media (**Figures 3D, E**). Together, these results suggest that cellular cGAS activity is required for maintaining cell survival during nutrient depletion.

### RU.521 induces autophagy-mediated cytotoxicity in serum-depleted media

**Figure 4:**
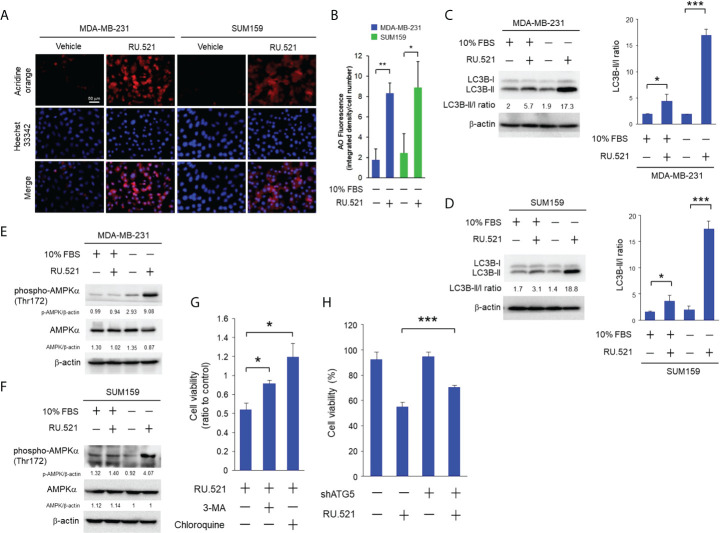
RU.521 induced cell death by autophagy in TNBC cells cultured in serum-depleted media. **(A)**, MDA-MB-231 and SUM159 cells were treated with RU.521 at 10 μM and 8 μM, respectively, in serum-depleted media for 48 hr. Cells were then stained with acridine orange and the red fluorescence signaling were captured by fluorescence microscopy at 400x magnification. Bar, 50 μm. The nuclei were counterstained by Hoechst 33342 (blue). **(B)**, The relative fluorescence intensity was quantitated. The data of three independent biological repeats are shown as means ± SD. *, p < 0.05; **, p < 0.01. Statistical significance was calculated by Student’s t-test. **(C, D)**, Expression of the LC3B-l and LC3B-ll proteins in MDA-MB-231 **(C)** and SUM159 **(D)** was determined by Western blotting analysis with an anti-LC3B antibody (upper panels). β-actin expression was served as a loading control. For each cell line, the relative fluorescence intensities were quantitated (lower panels). The data of three independent biological repeats are shown as means ± SD. *, p < 0.05; ***, p < 0.001. Statistical significance was calculated by Student’s t-test. **(E, F)** Expression of total AMPK and pT172-AMPK in MDA-MB-231 **(E)** and SUM159 **(F)** cultured in the presence and absence of 10% FBS and RU.521 were analyzed by Western blotting analysis with the corresponding antibodies. β-actin expression was served as a loading control. **(G)**, MDA-MB-231 cells were pre-treated with 3-MA (5 mM) or chloroquine (2 μM) in serum-depleted medium for 1 hr, followed by treatment with RU.521 (8 μM) for 24 hr. **(H)**, ATG5 expression of MDA-MB-231 cells was down-regulated by an ATG5-specific shRNA, followed by treatment with RU.521 (8 μM) in serum-depleted media for 24 hr. Cell growth was determined by crystal violet staining and detected by absorbance at OD 595 nm. The data from three independent biological repeats were plotted and presented as means ± SD. Statistical significance was calculated by Student’s t-test. ***, p < 0.001.

Autophagy can lead to cell death or assist survival pending on the biological context (16). Autophagy accompanied by intracellular hypervacuolization has been shown to promote cell death instead of survival (15). RU.521 treatment resulted in reduced cell viability accompanied by the accumulation of cytoplasmic vesicles in cells cultured in serum-depleted media (**Figure 3A**). Thus, the vacuolar phenotype of the cells dying from cGAS inhibition under growth-restricted condition suggests an autophagy-dependent mechanism of the cell death. In consistence with this speculation, staining with acridine orange, an acid-resistant fluorescent dye which can withstand acidic organelles such as autophagosome and emits red fluorescence (17), showed prominent acidic signals in the cytoplasm of serum-starved MDA-MB-231 and SUM159 cells in the presence RU.521 for 48 h (**Figures 4A, B**). The induction of autophagy was confirmed by the increased LC3B-II to LC3B-I ratios in cells cultured in serum-depleted media and treated with RU.521 (**Figures 4C, D**) (18). Similarly, RU.521 induced autophagy of MDA-MB-231 and SUM159 cells cultured in 1% serum (**Supplementary Figures 4C, D**). And the LC3B-II to LC3B-I rations of MDA-MB-231 and SUM159 cells were increased under 1% serum in the presence RU.521 (**Supplementary Figures 4E, F**). In drastic contrast, treatment with RU.521 alone had only marginal effect on LC3B-II/LC3B-I ratios in cells grown in normal media (**Figures 4C, D**). A major mechanism of autophagy stimulation is through the energy-sensing pathway moderated by the protein kinase AMPK (19). Upon treatment with RU.521 in serum-depleted media, AMPK activation as indicated by the phosphorylation of AMPKα at Thr172 was increased (**Figures 4E, F**). AMPK activation leads to the stimulation of the class III PI3K in the Beclin complex and autophagosome initiation (20). Treatment by 3-MA, an inhibitor of the class III PI3K (21), and chloroquine, an inhibitor of autophagolysosome maturation (22), significantly rescued cell death (**Figure 4G**). The causal relationship of autophagy was further confirmed by depleting ATG5, an autophagic gene essential for autophagosome formation (23), which rescued cell survival (**Figure 4H**). On the other hand, treatments with inhibitors of apoptosis [z-VAD (24)], necrosis [necrostatin-1 (25)], ferroptosis [ferrostatin-1 (26)] did not affect cell viability (**Supplementary Figure 5**). These results together demonstrate that the cell death upon cGAS inhibition in serum-depleted media was mainly driven by autophagy.

**Figure 5:**
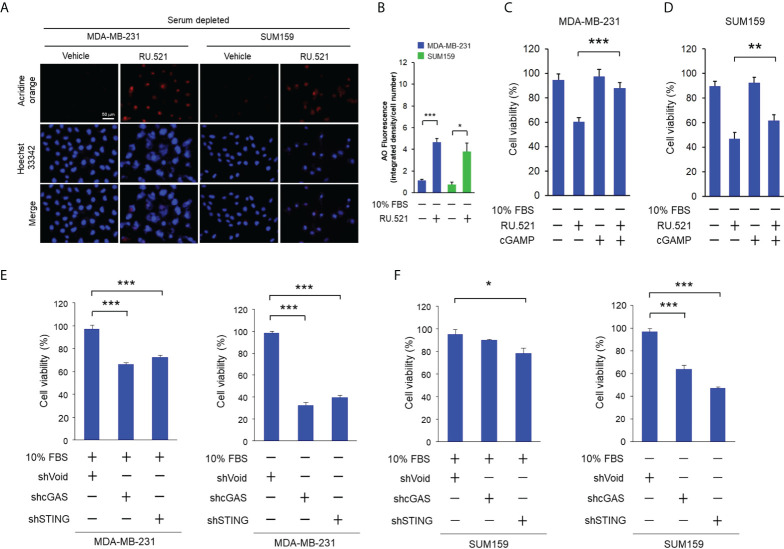
Stimulation of the cGAS-STING pathway rescued growth suppression by RU.521 under serum-depleted condition. **(A)**, MDA-MB-231 and SUM159 cells were treated with RU.521 at 10 μM and 8 μM, respectively, in serum-depleted media for 24 hr. Cells were then stained with acridine orange and red fluorescence was captured by fluorescence microscopy at 400x magnification. The nuclei were counterstained by Hoechst 33342 (blue). Bar, 50 μm. **(B)**, The images of relative fluorescence intensity were quantitated and plotted. The data from three independent biological repeats were plotted and presented as means ± SD. *, p < 0.05; ***, p < 0.001. Statistical significance was calculated by Student’s t-test. **(C, D)** MDA-MB-231 **(C)** and SUM159 **(D)** were transiently transfected with cGAMP (10 μM) by lipofectamine 2000, followed by treatment with RU.521 (8 μM) in serum-free media for 24 hr. Cell viability was measured by crystal violet staining and detected by absorbance at 595 nm. **(E, F)** MDA-MB-231 **(E)** and SUM159 **(F)** cells were infected by lenti-viral vectors expressing the shRNA against cGAS (shGAS), STING (shSTING), or the control vector, followed by culturing in serum-replete (+10% FBS) or serum-depleted (-10% FBS) media as indicated. Cell viability was measured by crystal violet staining and detected by absorbance at 595 nm. In each experiment, the data of three independent biological repeats are shown as means ± SD. *, p < 0.05; **, p < 0.01; ***, p < 0.001. Statistical significance was calculated by Student’s t-test.

These results suggest that activation of the cGAS-STING pathway protects cells from serum depletion. If this is the case, introduction of the cGAMP dinucleotide, the secondary messenger produced by cGAS and the activator of STING (1, 27), should rescue the survival of cells treated with RU.521 in serum-depleted media. To test this hypothesis, cGAMP was introduced to cells by transient transfection. It should be noted that considering the transient nature of this experiment, cells were cultured for 24 hr after transfection. The induction of autophagy in this time window was confirmed by acridine staining showing the presence of autophagic intracellular vesicles (**Figures 5A, B**). Transient transfection of cGAMP significantly rescued the viability of MDA-MB-231 treated with RU.521 in serum-depleted media (**Figure 5C**). Similar effect was observed in the case of SUM159 albeit to a less extent likely due to different cellular contexts (**Figure 5D**). These results together support that the growth suppression effect by RU.521 was driven by its cGAS-inhibiting activity. To further verify the role of the cGAS-STING pathway in maintaining cell viability in growth-restricted condition, endogenous cGAS and STING were depleted by specific short hairpin RNAs of cGAS (shcGAS) and STING (shSTING), respectively, in MDA-MB-231 and SUM159 cells (**Supplementary Figure 6**). Down-regulation of cGAS or STING only resulted in marginal growth inhibition in serum-replete media in MDA-MB-231 (**Figure 5E**) and SUM159 (**Figure 5F**) cells. In drastic contrast, downregulating cGAS or STING led to significant reduction of cell viability for both cell lines cultured in serum-depleted condition (**Figures 5E, F**). These results together demonstrate a function of the cGAS-STING pathway in maintaining cell viability when confronted by growth-restricted conditions.

Intriguingly, treatment of MDA-MB-231 and SUM159 cells cultured in serum-replete media with cGAMP also resulted in prominent enhancement of autophagy as demonstrated by the acridine-positive intracellular vesicles (**Supplementary Figures 7A–D**). In contrast, treatment with RU.521 alone only resulted in slight increase of autophagy in serum-replete media (**Figures 4C, D**; **Supplementary Figures 7A–D**). Consisting with the fact that cGAMP activates STING as a downstream event of the cGAS activity, co-treatment with RU.521 did not further enhance the level of autophagy in the presence of cGAMP. As shown above, treatment of the two cell lines cultured in serum-depleted media with RU.521 resulted in significant autophagy and cell death (**Figures 3**–**5**; **Supplementary Figures 7E, F**). In contrast, the autophagy induced by cGAMP in serum-replete media was not associated with cell death (**Supplementary Figures 7E, F**).

### Co-inhibition of cGAS and receptor tyrosine kinase (RTK) results in cell killing

**Figure 6:**
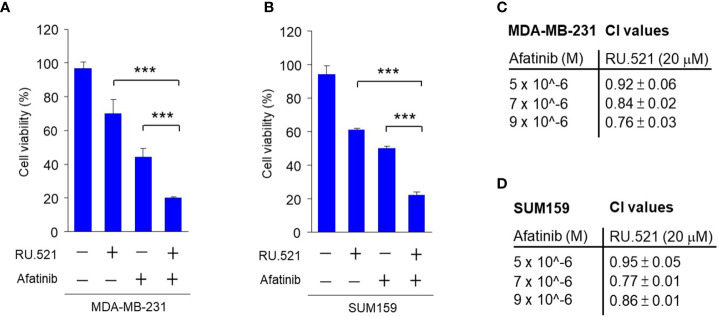
The synergistic effect in inhibiting the viability of TNBC cells by combining RU.521 and afatinib in serum-replete media. A and B, MDA-MB-231 **(A)** and SUM159 **(B)** cells were treated with RU.521 (20 μM) or afatinib (5 μM) alone, and combined treatment with RU.521 plus afatinib in normal growth media for 48 hr. Cell viability was determined by crystal violet staining and detected by absorbance at OD595 nm. The data from three independent biological repeats data are plotted and presented as means ± SD. Statistical significance was calculated by Student’s t-test. ***, p < 0.001. C and D, MDA-MB-231 **(C)** and SUM159 **(D)** cells were treated with and without RU.521 (20 μM) in the presence of different concentrations of afatinib (0, 5, 7, 9 μM) for 48 hr. Cell viability was determined as described in **(A)** and **(B)** The data were analyzed by the formula of CI=(D)_1_/(Dx)_1_+(D)_2_/(Dx)_2,_ in which a CI < 1 suggests synergism.

These results also led to the speculation that the growth factors present in the serum can protect cells from the cell-killing effect of RU.521, and that concomitantly blocking the growth signaling in cells cultured in normal media in the presence of RU.521 should achieve the same cytotoxic effect as serum depletion in the presence of RU.521. EGFR is a major RTK expressed in MDA-MB-231 and SUM159 cells and promotes their growth (28, 29). In serum-replete media, treatment with RU.521 or the irreversible EGFR inhibitor afatinib alone resulted in moderate cell toxicity, while combined treatment of RU.521 with afatinib caused a prominent cell toxicity in MDA-MB-231 and SUM159 cells (**Figures 6A, B**). And combined treatment of RU.521 with different doses of afatinib in MDA-MB-231 and SUM159 cells resulted in combination index (CI) values less than 1 (**Figures 6C, D**), suggesting a synergistic cell toxicity based on isobologram analyses (11). Thus, these results suggest a collaborative relationship of the cGAS and EGFR pathways in supporting tumor growth.

**Figure 7:**
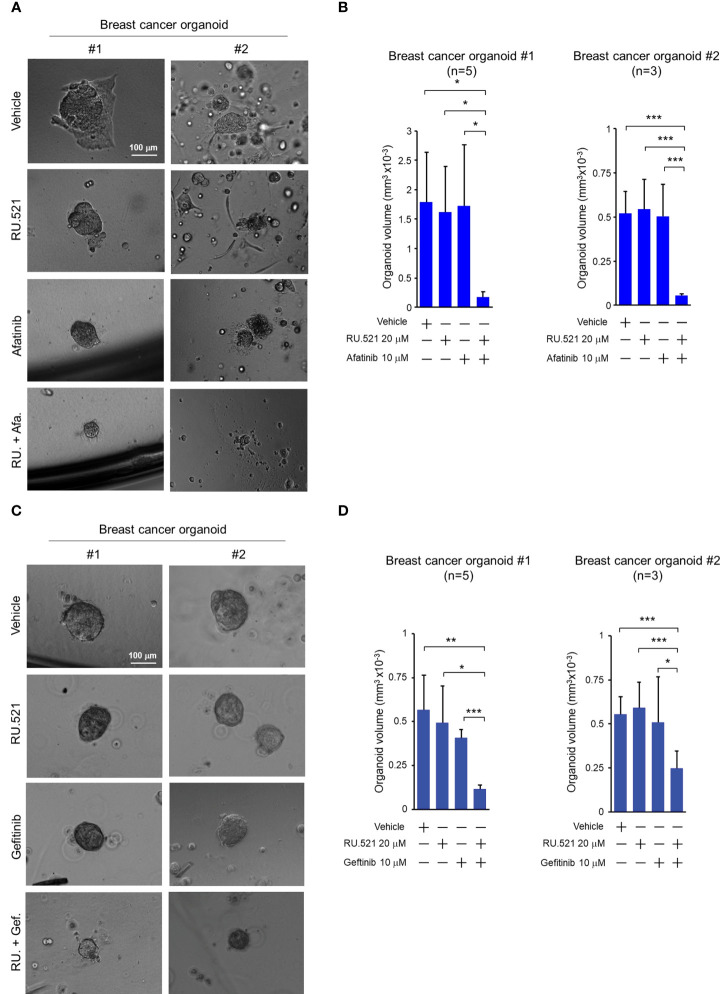
Combined treatment by RU.521 and EGFR inhibitors suppressed the growth of tumor organoids derived from breast cancer patients. Tumor organoids derived from breast cancer patients (#1 and #2) were cultured and treated with RU.521 alone (20 μM), EGFR inhibitors (afatinib (A) or gefitinib (C)) alone (10 μM), or co-treated with RU.521 plus an EGFR inhibitors (afatinib (A) or gefitinib (C)) in serum-replete media for 72 hr. **(A, C)** Representative images of organoids captured by an inverted microscope at 100x magnification are shown. Bar, 100 μm. **(B, D)** the volumes of the derived organoids (n=5 of #1, n=3 of #2 for each treatment) after the indicated treatments were estimated by the sphere volume formula (V=4/3πr3). The data of three independent experiments were shown as means ± SD, and statistical significance was plotted by Student’s t-test. *, p < 0.05; **, p < 0.01; ***, p < 0.001.

**Figure 8:**
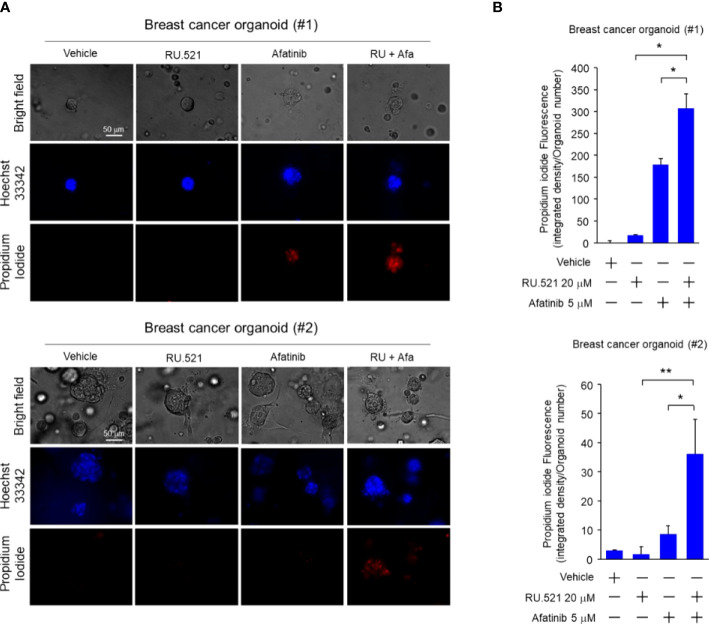
Combined treatment by RU.521 and afatinib induced death of tumor cells of the PDTOs. The tumor organoids derived from breast cancer patients were cultured and treated with RU.521 alone (20 μM), afatinib alone (5 μM), or co-treated with RU.521 plus afatinib in serum-replete media for 72 hr. n, the number of organoids tested in each treatment are indicated. **(A)**, The organoids were stained by Hoechest 33342 and propidium iodide for 1 hr, and fluorescent images were captured by a fluorescence microscope at 400x magnification. The images of representative organoids including the corresponding bright field images are shown. **(B)**, The fluorescence intensities of the organoids were quantitated and plotted. The data of three independent experiments were shown as means ± SD, and statistical significance was plotted by Student’s t-test. *, p < 0.05; **, p < 0.01.

Increased EGFR expression is frequently found in breast cancer including TNBC (30–32). Patient-derived tumor organoids (PDTOs) were generated from two breast cancer patients. The organoids were mock-treated or treated with RU.521 alone, afatinib alone, gefitinib alone, RU.521 plus afatinib, or RU.521 plus gefitinib. Organoid growth as measured by the sizes showed that the single treatments had no effect to the growth of the organoids. In contrast, combined treatment of RU.521 and afatinib (**Figures 7A, B**), or RU.521 combined with gefitinib (**Figures 7C, D**) dramatically suppressed organoid growth. To further define the mechanism of organoid suppression, the PDTOs from the same patients were mock-treated or treated with RU.521 alone, afatinib alone, or RU.521 plus afatinib, followed by staining with Hoeschst 33342 and propidium iodide. Hoeschst 33342 promiscuously stains the nuclei while propidium iodide stains dead cells. Fluorescent imaging of the organoids showed that the combined treated significantly killed tumor cells compared to the single treatments (**Figure 8**). These results together support the therapeutic potential of combined inhibition of the cGAS-STING and RTK pathways for breast cancer.

## Discussion

Our results demonstrate an unexpected function of the stress sensor cGAS under a growth-limiting serum-depleted condition. Deregulated proliferative DNA synthesis renders cancer cells continuously confronted by the challenge of genomic instability. In the current study, we show that the ssDNA byproduct derived from the aberrant genome metabolism is exploited by cancer cells to maintain viability under stressed conditions. The cGAS sensor for cytosolic DNA has been shown as an important mechanism to forge immune responses to invading pathogens and the surveillance for cell transformation, thus stimulation of the cGAS-STING pathway has been traditionally viewed as an anti-tumor measure which has been intensively exploited for therapeutic gains (33). Opposing to this traditional conception, our study suggests that the intrinsic function of cGAS-STING can also play an important role in sustaining viability of cancer cells in hostile growth condition. Chronical activation of the cGAS-STING-TBK1 pathway promotes inflammation-driven tumorigenesis (34, 35). More recently, it was reported that the tumor-derived cGAMP can induce astrocytes to produce metastasis-promoting inflammatory cytokines (36). In addition, activation of STING can enhance tumor growth through upregulation of the immunosuppressive dioxygenase IDO (37). Together these studies highlight the novel finding of the cGAS-STING pathway in stimulating tumor progression instead of suppression.

Our results demonstrate that the starvation-induced cGAS-STING axis protects cells from autophagy-mediated cell death. Intriguingly, transfection of cGAMP to MDA-MB-231 and SUM159 cells cultured in serum-replete media resulted in prominent enhancement of autophagy as demonstrated by the acridine-positive intracellular vesicles. It has been reported that activation of the cGAS-STING pathway can also lead to the induction of autophagy under different biological conditions. Stimulation of the cGAS-STING pathway by cytosolic viral DNA is a primordial mechanism of inducing autophagy flux to clear intracellular virus, forming the fundamental biology for cells to fending off viral infection (38). Nassour et al. reported that telomere dysfunction in cells experiencing replicative crisis triggers activation of the cGAS-STING axis, which in turn engages the autophagic cell death as a final defense against cell transformation (39). In contrast, our results show that inhibition of the cGAS-STING axis in serum-depleted condition results in cell death and tumor growth suppression. Thus, activation of the cGAS-STING axis can result in promotion or inhibition of autophagy which may or may not be associated with cell viability depending on the culturing conditions. Together, these independent studies highlight the complexity of autophagic response triggered by the activation of the cytosolic pattern recognition machineries for which the molecular basis of the functional consequences remains to be determined. These results also imply that the crosstalk between the cGAS-STING cascade and autophagy regulation plays an important role in responding to cellular stresses of both intrinsic and extrinsic origins. Further studies are needed to dissect the molecular mechanisms underlying the distinct biological consequences in different cellular contexts.

Further studies are also needed to determine the mechanisms of directing the cGAS-STING activation to anti- or pro-tumor autophagic flux. It has been suggested that the bifurcation of cell death and growth downstream of autophagy highly depends on the cellular context (40). The autophagic flux induced by nutrient withdrawal is regarded in general to be promoting cell survival. On the other hand, autophagy induced by oncogenes such as Ras can lead to either cell death or survival depending on the molecular context in the cancer cells (41, 42). Our data demonstrate that TBK1 was activated in cells cultured in serum-depleted media, likely due to induction by the cytosolic ssDNA. It has been shown that TBK1 activation by the cGAS-STING pathway suppresses AMPK (43), a critical energy sensor for autophagy stimulation (44). Consistently, treatment of the serum-starved cells with RU.521 on one hand blocked TBK1 (**Supplementary Figure 2**), and on the other hand activated AMPK (**Figures 4E, F**), hence promoted autophagy progress. In the current study, we show that growth factor starvation alone did not induce autophagy or any form of cell death until co-treatment of RU.521 which triggered massive cell death through autophagy. These results suggested that cGAS, by working together with survival signaling such as the tyrosine kinase EGFR, plays an important function for cancer cells to survive sustained growth stresses.

It is noted that RU.521 treatment slightly induced autophagy in cells under nutrient-replete condition as observed by LC3 lipidation when activation of the energy sensor AMPK was not detected. Autophagy is known to be regulated by both canonical and non-canonical pathways and numerous AMPK-independent mechanisms, such as the calcium/calmodulin-dependent protein kinases and the MAPK kinases, have been shown to regulate autophagy in a context-dependent manner (45). It remains to be determined whether and how RU.521 treatment regulates the development of autophagy in different energy contexts.

The current work has focused on TNBC tumors. TNBC accounts for 10%-20% of breast cancer and characterized by the lack of traditional therapeutic targets, including estrogen receptor, progesterone receptor, and ErbB-2/HER-2/*neu*. Thus, developing targeted therapy for TNBC has been a major challenge and chemotherapy remains to be the mainstay therapy for TNBC (46). In general, TNBC tumors are more aggressive with shorter time to relapse following the frontline therapy than other types of breast cancer. EGFR expression is a frequent event in TNBC (13-78%), making it a promising TNBC marker and potential target (47). However, the efficacy of targeting EGFR as single therapy in breast cancer has been disappointing. Our study shows that anti-EGFR regimens combined with targeting the cytosolic ssDNA-cGAS-STING axis may be a promising therapeutic opportunity to overcome TNBC.

The current study employs patient-derived tumor organoids (PDTOs) to test the combined inhibition of cGAS-STING and EGFR. PDTOs is emerging as a promising *in vitro* model for human diseases (48). It preserves the major cellular, histological, and genetic contexts of the normal or diseased tissues such as cancer and can be generated in a timely manner compared to the relatively long latency patient-derived xenograft (PDX) model (49). For human breast cancer, it has been established that PDTOs is a promising model system to recapitulate the pathology of advanced tumors and can be leveraged for assessing the predicted response of the primary tumors to pharmacological therapeutics (12, 50, 51). In the current study, treatment by the combination of RU.521 and EGFR inhibitors (afatinib or gefitinib) on two independent PDTOs supports the tumor-suppressing activity of co-inhibition of the cGAS-STING and EGFR pathways. The results provide a proof-of-concept for the combined therapy and a potential platform to pre-assess the clinical response to the therapy.

Overall, the current study unveils an unexpected function of the cGAS-STING axis in promoting cancer cell survival and demonstrates the potential of developing the stress-responding pathway as a therapeutic target. Given the finding of the cGAS-STING-mediated growth advantage, our results highlight the potential complications of the popular approaches to enhance the activity of the cGAS-STING axis as a means of anti-cancer therapy.

## Data availability statement

The original contributions presented in the study are included in the article/**Supplementary Material**. Further inquiries can be directed to the corresponding authors.

## Ethics statement

The studies involving human participants were reviewed and approved by the research ethics committee of China Medical University and Hospital (approval number, CMUH105-REC1-064(CR-5)). The patients/participants provided their written informed consent to participate in this study.

## Author contributions

S-CW conceived the project, S-CW, C-CL, and L-CL designed the experiments, analyzed the data, coordinated the collaborating groups; L-CL surgically collected the tumor tissues from breast cancer patients following the approved institutional IRB protocol; C-CL performed most experiments and data collection with the help of W-RW; Y-CS performed the immunostaining experiments; Y-LW developed the organoid culture; L-CC assisted the experiments discerning cell death mechanisms; Y-HC helped with experimental design and manuscript preparation; S-CW wrote the original draft of the manuscript; S-CW and C-CL prepared the final manuscript. All authors contributed to the article and approved the submitted version.

## Funding

This study was supported in part by the National Health Research Institute (NHRI-EX110-11012BI), the Ministry of Science and Technology (MOST-111-2314-B-039 -055-MY3, MOST-111-2314-B-039-030), the Ministry of Health and Welfare (MOHW107-TDU-B-212-112015), the China Medical University Hospital (grant DMR-CELL-17025), the China Medical University Research (Grant CMU107-TU-01) (to S-CW). This work was also financially supported in part by the Drug Development Center of the China Medical University from The Featured Areas Research Center Program within the framework of the Higher Education Sprout Project by the Ministry of Education in Taiwan (to S-CW). Experiments and data analysis were supported by the Medical Research Core Facilities Center under the Office of Research & Development of CMU.

## Conflict of interest

The authors declare that the research was conducted in the absence of any commercial or financial relationships that could be construed as a potential conflict of interest.

## Publisher’s note

All claims expressed in this article are solely those of the authors and do not necessarily represent those of their affiliated organizations, or those of the publisher, the editors and the reviewers. Any product that may be evaluated in this article, or claim that may be made by its manufacturer, is not guaranteed or endorsed by the publisher.

## Supplementary material

Supplementary Figure 1 Expression of endogenous cGAS under normal and serum-depleted media. MDA-MB-231 **(A)** and SUM159 **(B)** cells cultured in media with and without 10% FBS for 24 hr were harvested and the cell lysates were subject to Western blotting analysis using the cGAS, STING and β-actin antibodies.

Supplementary Figure 2 Activation of the cGAS-STING-cytokine pathway by serum depletion. MDA-MB-231 cells were cultured in serum-replete or serum-depleted media for 24 hr. Cells were harvested and the lysates were analyzed by Western blotting analysis using the indicated antibodies.

Supplementary Figure 3 Serum depletion induced accumulation of cytosolic ssDNA in TNBC cells. **(A)** MDA-MB-231 and SUM159 cells were cultured in normal or serum-depleted media for 48 hr. The production of ssDNA expression was detected by OliGreen staining. The nuclei were counterstained with Hoechst 3342. Fluorescence images were captured at 400x magnification. Bar, 50 μm. **(B)**, The images in A were quantitated and plotted. The data of three independent results are shown as means ± SD and the statistical significance was calculated by Student’s test. *, p < 0.05; **, p < 0.01. **(C)**, Cells cultured in conditions as described in A were fixed and stained with PicoGreen for double-strand DNA. The nuclei were counterstained with Hoechst 3342. Fluorescence images were captured at 400x magnification. **(D)**, The images in C were quantitated and plotted. The data of three independent results are shown as means ± SD and the statistical significance was calculated by Student’s test. NS, non-significant. **(E)**, Cells cultured in conditions as described in A were fixed and stained with propidium iodide (PI)/BrdU, and the cell cycle profiles were analyzed by flow cytometry. The numbers of cells in Sub-G1, G1, S and G2/M were indicated with corresponding standard error. **(F)**, The data of three independent biological repeats of cell cycle profiling as described in E were plotted with means ± SD. Statistical significance was calculated by Student’s t test. NS, non-significant; *, p < 0.05; **, p < 0.01; ***, p < 0.001. **(G)**, Cells cultured in conditions as described in A were stained with 7-AAD and a FITC-conjugated anti-annexin V antibody, and the apoptotic cell population was assessed by flow cytometry. The percentage of apoptotic cells was indicated. **(H)**, The data derived from three independent experiments as described in G were plotted as means ± SD and the statistical significance was calculated by Student’s t test. *, p < 0.05.

Supplementary Figure 4 Low (1%) serum induced cytosolic ssDNA accumulation and promoted RU.521-induced autophagy in TNBC cells. **(A)**, MDA-MB-231 and SUM159 cells were cultured in media containing normal (10%) or low (1%) FBS for 48 hr. The production of ssDNA expression was detected by OliGreen staining. The nuclei were counterstained with Hoechst 3342. Fluorescence images were captured at 400x magnification. Bar, 50 μm. **(B)**, The images in A were quantitated and plotted. The data of three independent repeats are shown as means ± SD and the statistical significance was calculated by Student’s test. *, p < 0.05. **(C)**, MDA-MB-231 and SUM159 cells were treated with RU.521 at 10 μM and 8 μM, respectively, in low serum (1% FBS) media for 48 hr. Cells were then stained with acridine orange and red fluorescence was captured by fluorescence microscopy at 400x magnification. The nuclei were counterstained by Hoechst 33342 (blue). Bar, 50 μm. **(D)**, The relative fluorescence intensities were quantitated. The data of three independent biological repeats are shown as means ± SD. *, p < 0.05. Statistical significance was calculated by Student’s t-test. **(E, F)**, Expression of the LC3B-l and LC3B-ll proteins in MDA-MB-231 **(E)** and SUM159 **(F)** was determined by Western blotting analysis using an anti-LC3B antibody (upper panels). β-actin expression was used as a loading control. For each cell line, the relative fluorescence intensities were quantitated (lower panels). The data of three independent biological repeats are shown as means ± SD. ***, p < 0.001. Statistical significance was calculated by Student’s t-test.

Supplementary Figure 5 RU.521 caused cell death through mechanisms independent of apoptosis, necrosis, or ferroptosis. MDA-MB-231 cells were pre-treated with different inhibitors (z-VAD, 100 μM; necrostatin-1, 100 μM; ferrostatin-1, 2 μM) for 1 hr, followed by co-treatment with or without RU.521 (10 μM) in serum-free media for 24 hr. Cell viability was determined by crystal violet staining for absorbance at OD595 nm. Data from three independent biological repeats are plotted and presented as means ± SD. Student’s t test showed statistical insignificance with p > 0.05.

Supplementary Figure 6 The knockdown efficiency of the endogenous cGAS and STING expression by shRNA. MDA-MB-231 **(A)** and SUM159 **(B)** cells grown in normal media were infected with lenti-viral vectors of shRNA against cGAS (shGAS) and STING (shSTING). Western blotting analyses were performed using the indicated antibodies. The expression of β-actin was used as the internal control.

Supplementary Figure 7 The cGAS-STING pathway differentially modulates autophagic response in different culturing conditions. **(A, C)**, MDA-MB-231 **(A)** and SUM159 **(C)** cells were transiently transfected with cGAMP (10 μM) by lipofectamine 2000, followed by treated with RU.521 at 10 μM and 8 μM, respectively, in serum-replete media for 24 hr. For comparison, the cells of both cell lines were treated with RU.521 under serum-depleted media for 24 hr. Cells were then stained with acridine orange and red fluorescence was captured by fluorescence microscopy at a magnification of 400x. Bar, 50 μm. The nuclei were counterstained by Hoechst 33342 (blue). **(B, D)**, The images in **(A, C)**, respectively, were quantitated and plotted. The data of three independent biological repeats are shown as means ± SD and the statistical significance was calculated by Student’s test. *, p < 0.05; **, p < 0.01; ***, p < 0.0001. **(E, F)**, MDA-MB-231 (E) and SUM159 (F) cells were treated in the conditions as described in **(A, C)**, and cell viability was determined by staining with crystal violet which was then measured by absorbance at OD595 nm. The data of three independent biological repeats are shown as means ± SD. *, p < 0.05; ***, p < 0.001. Statistical significance was calculated by Student’s t-test.
