# Supplementary figures and images for: Growth-promoting function of the cGAS-STING pathway in triple-negative breast cancer cells
Source: Front Oncol. 2022 Aug 3;12:851795. doi: 10.3389/fonc.2022.851795 (PMC9385397; doi:10.3389/fonc.2022.851795)

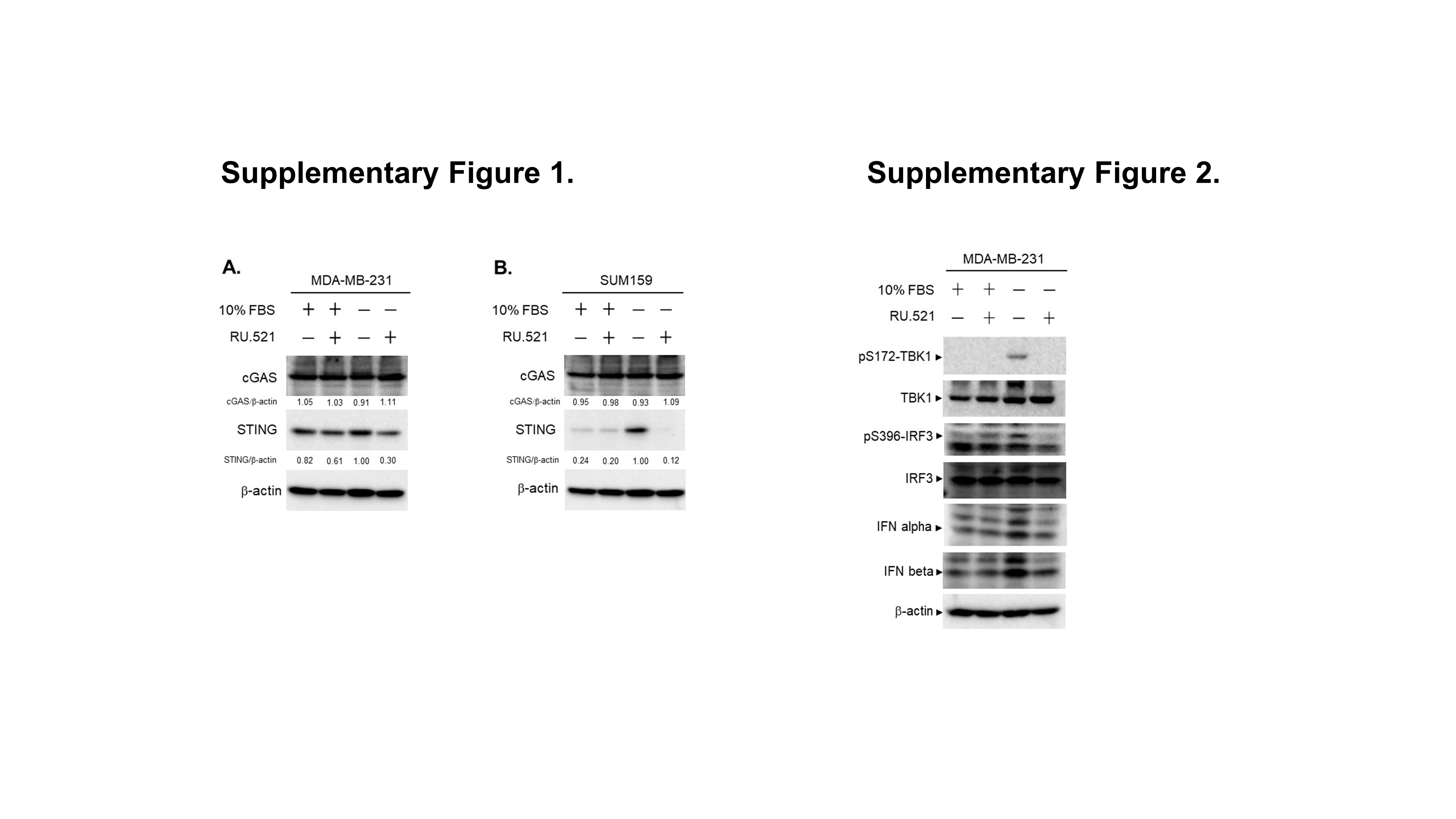

Supplement: Supplementary Figure 1 — Expression of endogenous cGAS under normal and serum-depleted media. MDA-MB-231 (A) and SUM159 (B) cells cultured in media with and without 10% FBS for 24 hr were harvested and the cell lysates were subject to Western blotting analysis using the cGAS, STING and β-actin antibodies. [file Image_1.tif]

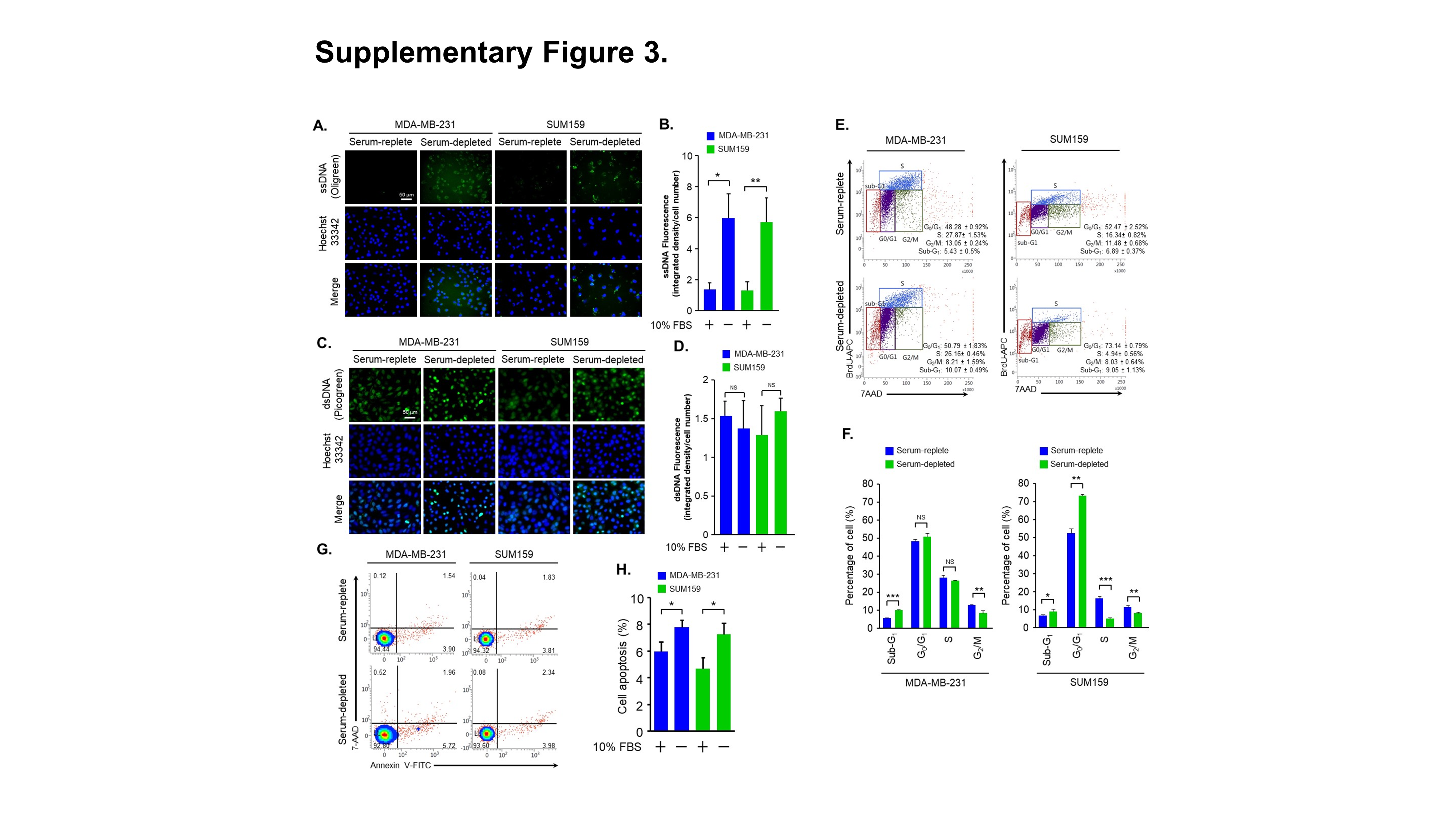

Supplement: Supplementary Figure 3 — Serum depletion induced accumulation of cytosolic ssDNA in TNBC cells. (A) MDA-MB-231 and SUM159 cells were cultured in normal or serum-depleted media for 48 hr. The production of ssDNA expression was detected by OliGreen staining. The nuclei were counterstained with Hoechst 3342. Fluorescence images were captured at 400x magnification. Bar, 50 μm. (B), The images in A were quantitated and plotted. The data of three independent results are shown as means ± SD and the statistical significance was calculated by Student’s test. *, p < 0.05; **, p < 0.01. (C), Cells cultured in conditions as described in A were fixed and stained with PicoGreen for double-strand DNA. The nuclei were counterstained with Hoechst 3342. Fluorescence images were captured at 400x magnification. (D), The images in C were quantitated and plotted. The data of three independent results are shown as means ± SD and the statistical significance was calculated by Student’s test. NS, non-significant. (E), Cells cultured in conditions as described in A were fixed and stained with propidium iodide (PI)/BrdU, and the cell cycle profiles were analyzed by flow cytometry. The numbers of cells in Sub-G1, G1, S and G2/M were indicated with corresponding standard error. (F), The data of three independent biological repeats of cell cycle profiling as described in E were plotted with means ± SD. Statistical significance was calculated by Student’s t test. NS, non-significant; *, p < 0.05; **, p < 0.01; ***, p < 0.001. (G), Cells cultured in conditions as described in A were stained with 7-AAD and a FITC-conjugated anti-annexin V antibody, and the apoptotic cell population was assessed by flow cytometry. The percentage of apoptotic cells was indicated. (H), The data derived from three independent experiments as described in G were plotted as means ± SD and the statistical significance was calculated by Student’s t test. *, p < 0.05. [file Image_2.tif]

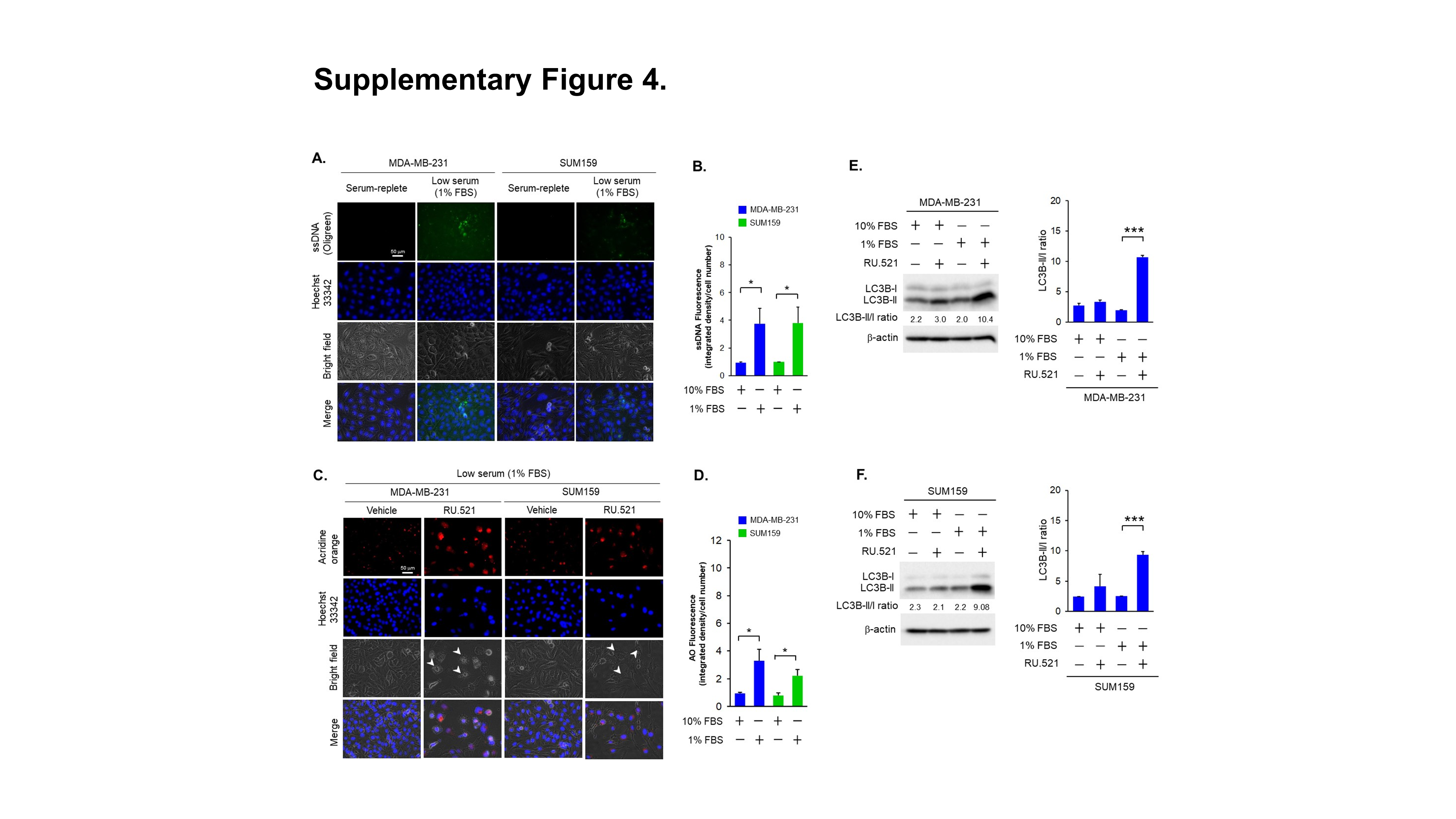

Supplement: Supplementary Figure 4 — Low (1%) serum induced cytosolic ssDNA accumulation and promoted RU.521-induced autophagy in TNBC cells. (A), MDA-MB-231 and SUM159 cells were cultured in media containing normal (10%) or low (1%) FBS for 48 hr. The production of ssDNA expression was detected by OliGreen staining. The nuclei were counterstained with Hoechst 3342. Fluorescence images were captured at 400x magnification. Bar, 50 μm. (B), The images in A were quantitated and plotted. The data of three independent repeats are shown as means ± SD and the statistical significance was calculated by Student’s test. *, p < 0.05. (C), MDA-MB-231 and SUM159 cells were treated with RU.521 at 10 μM and 8 μM, respectively, in low serum (1% FBS) media for 48 hr. Cells were then stained with acridine orange and red fluorescence was captured by fluorescence microscopy at 400x magnification. The nuclei were counterstained by Hoechst 33342 (blue). Bar, 50 μm. (D), The relative fluorescence intensities were quantitated. The data of three independent biological repeats are shown as means ± SD. *, p < 0.05. Statistical significance was calculated by Student’s t-test. (E, F), Expression of the LC3B-l and LC3B-ll proteins in MDA-MB-231 (E) and SUM159 (F) was determined by Western blotting analysis using an anti-LC3B antibody (upper panels). β-actin expression was used as a loading control. For each cell line, the relative fluorescence intensities were quantitated (lower panels). The data of three independent biological repeats are shown as means ± SD. ***, p < 0.001. Statistical significance was calculated by Student’s t-test. [file Image_3.tif]

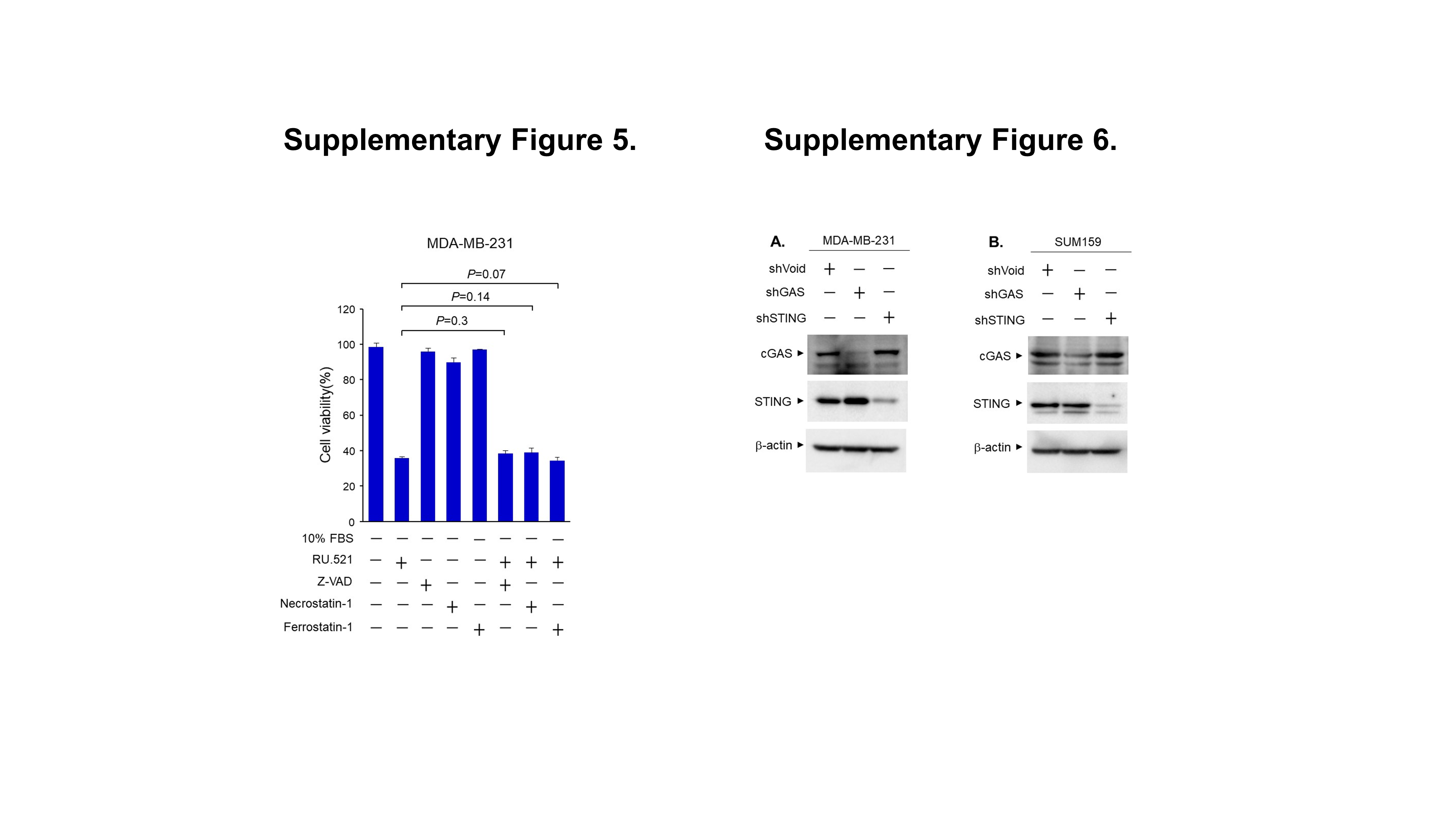

Supplement: Supplementary Figure 5 — RU.521 caused cell death through mechanisms independent of apoptosis, necrosis, or ferroptosis. MDA-MB-231 cells were pre-treated with different inhibitors (z-VAD, 100 μM; necrostatin-1, 100 μM; ferrostatin-1, 2 μM) for 1 hr, followed by co-treatment with or without RU.521 (10 μM) in serum-free media for 24 hr. Cell viability was determined by crystal violet staining for absorbance at OD595 nm. Data from three independent biological repeats are plotted and presented as means ± SD. Student’s t test showed statistical insignificance with p > 0.05. [file Image_4.tif]

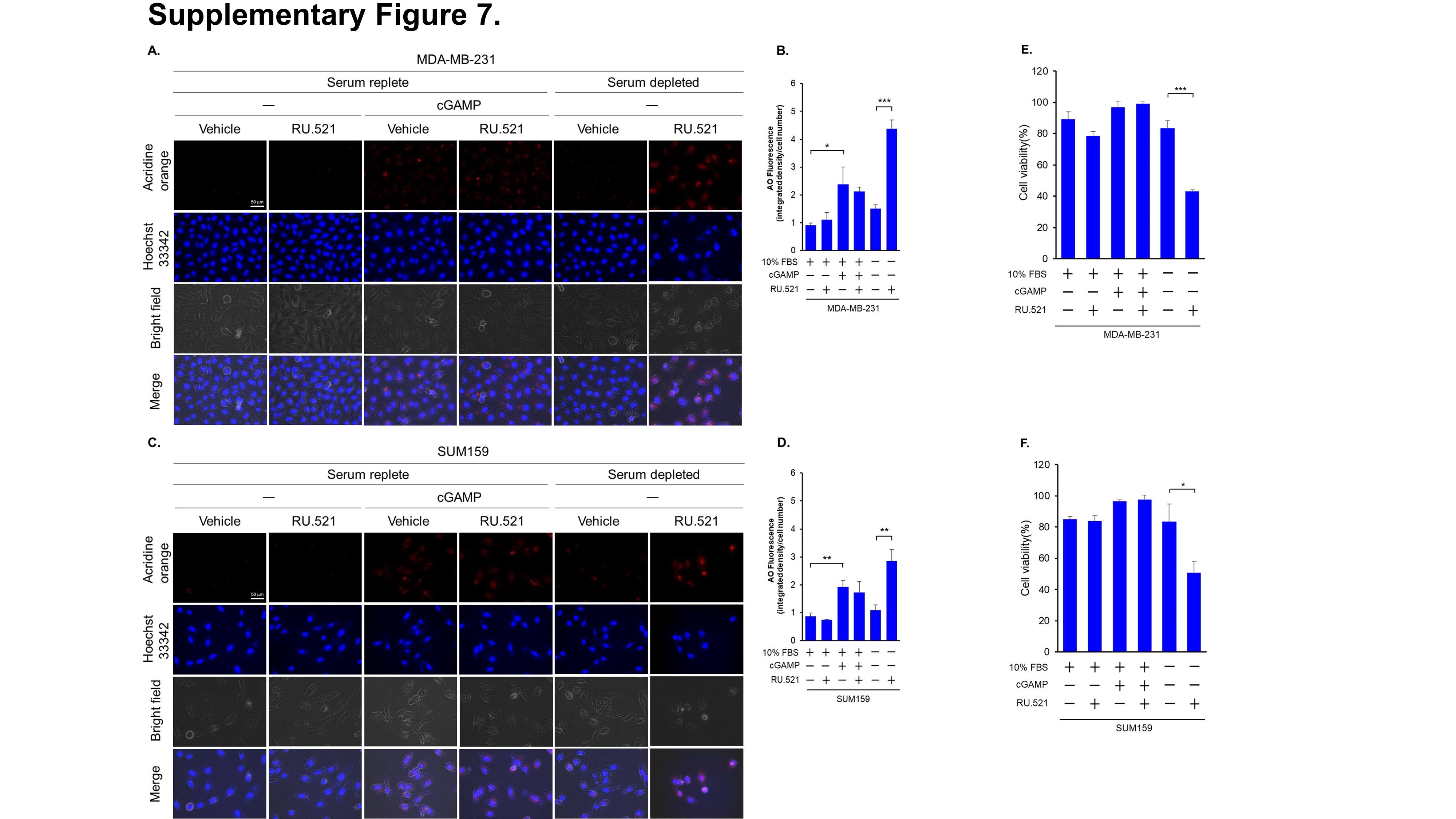

Supplement: Supplementary Figure 7 — The cGAS-STING pathway differentially modulates autophagic response in different culturing conditions. (A, C), MDA-MB-231 (A) and SUM159 (C) cells were transiently transfected with cGAMP (10 μM) by lipofectamine 2000, followed by treated with RU.521 at 10 μM and 8 μM, respectively, in serum-replete media for 24 hr. For comparison, the cells of both cell lines were treated with RU.521 under serum-depleted media for 24 hr. Cells were then stained with acridine orange and red fluorescence was captured by fluorescence microscopy at a magnification of 400x. Bar, 50 μm. The nuclei were counterstained by Hoechst 33342 (blue). (B, D), The images in (A, C), respectively, were quantitated and plotted. The data of three independent biological repeats are shown as means ± SD and the statistical significance was calculated by Student’s test. *, p < 0.05; **, p < 0.01; ***, p < 0.0001. (E, F), MDA-MB-231 (E) and SUM159 (F) cells were treated in the conditions as described in (A, C), and cell viability was determined by staining with crystal violet which was then measured by absorbance at OD595 nm. The data of three independent biological repeats are shown as means ± SD. *, p < 0.05; ***, p < 0.001. Statistical significance was calculated by Student’s t-test. [file Image_5.tif]
